# Long-Term Outcome and Athletic Level following Operative Treatment for Osteochondritis Dissecans of the Knee in Pediatric and Adolescent Patients

**DOI:** 10.3390/jcm12124140

**Published:** 2023-06-20

**Authors:** Yannic Bangert, Patrick Zarembowicz, Karoly Engelleiter, Evangelos Gkarilas, Holger Schmitt, Tobias Renkawitz, Ayham Jaber

**Affiliations:** 1 Department of Orthopaedics, Heidelberg University Hospital, 69118 Heidelberg, Germany; yannic.bangert@med.uni-heidelberg.de (Y.B.); tobias.renkawitz@med.uni-heidelberg.de (T.R.); 2 Department for Orthopaedic and Trauma Surgery, BG Klinik Ludwigshafen, 67071 Ludwigshafen am Rhein, Germany; patrick.zarembowicz@bgu-ludwigshafen.de; 3 Department for Orthopaedic and Trauma Surgery, Helios Clinic, 75175 Pforzheim, Germany; k.engelleiter@gmx.de; 4 Department for Orthopaedics, Trauma and Spinal Surgery, Neckar-Odenwald Clinics, 74821 Mosbach, Germany; ev.gkarilas@gmail.com; 5 German Joint Center, ATOS Clinic Heidelberg, 69115 Heidelberg, Germany; prof.hschmitt@googlemail.com

**Keywords:** osteochondritis dissecans, subchondral lesion, knee, adolescence, juvenile, autologous chondrocyte implantation

## Abstract

Research on the long-term outcomes following surgical therapy for osteochondritis dissecans (OCD) of the knee is scarce. A single-center retrospective cohort study was conducted to investigate surgically treated patients for knee OCD between 1993 and 2007. A total of 37 patients with an average follow-up duration of 14 years (range 8–18) were in the final cohort. IKDC and Lysholm scores were assessed. The duration and types of sport activity were reported. Long-term results were compared with existing midterm data. Knee scores showed a very good outcome with a mean of 91.3 in the IKDC score and 91.7 in the Lysholm score. Compared to midterm outcomes, both IKDC (*p* = 0.028) and Lysholm scores (*p* = 0.01) improved on final follow-up. Patients with open physes showed a significantly better Lysholm score compared to patients with closed physes (*p* = 0.034). Defect localization and size did not influence the outcome, but a defect depth of <0.8 cm^2^ achieved significantly better scores than ≥0.8 cm^2^. Of all surgical interventions, refixation achieved the best outcome. Long-term results significantly improved compared to midterm results with a follow-up of 40 months (*p* = 0.01). Thirty-six out of 37 patients were physically active, with 56% of sports being knee-straining activities. Long-term results following surgically treated OCD fragments show excellent function and a good athletic level. Patients with open physes potentially have better knee outcomes. Midterm results are sustainable and could improve further in the long term.

## 1. Introduction

The term osteochondritis dissecans (OCD) refers to the focal idiopathic alteration of the subchondral bone, which eventually leads to instability and the disruption of articular cartilage and results in premature osteoarthritis [1]. An unstable osteochondral fragment may result in a free body, as originally described by König in 1887 [2]. OCD could occur in any joint in the body, but the knee is most commonly involved [3]. It primarily affects adolescents with an incidence of 9.5 per 100,000 patients [4]. A prevalence is observed in athletes compared to nonathletes. In a large multicenter study, around 70% of all patients with OCD of the knee were multisport athletes, with the most common practiced sports being basketball for males and football for females [5].

The definitive etiology remains unclear. It is believed that mechanical stress in the sense of microtrauma or repetitive stress evokes a subchondral reaction that interferes with trabecular bone healing, preventing the bone’s ability to recover. This mechanism is currently believed to be the primary ethiology [6]. Furthermore, biological, hereditary, and anatomical factors are also speculated to play a role in the development of OCD [7]. Brown et al. showed a correlation between the location of OCD and lower limb mechanical axis deviation (varus/valgus) [8]. Moreover, complete discoid menisci were associated with central OCD, while incomplete discoid menisci were associated with peripheral OCD [9]. Biomechanical factors such as obesity and soft-tissue instability have also been implicated such as in the case of cruciate ligament hypoplasia [6].

The most common location of knee OCD is the distal femur, specifically in the lateral aspect of the medial femoral condyle (MFC) [10]. In a population-based cohort study of OCD of the knee in a pediatric population, male patients had a greater risk of OCD, with almost 4 times the incidence compared to female patients [11]. Ages 12 to 19 years exhibit around 3 times the risk of developing OCD of the knee compared to 6 to 11 year-old children [4]. The initial stages of the disease are often characterized by only mild symptoms such as knee swelling following significant activity [5]. In later stages, the osteochondral lesion becomes unstable and ultimately becomes a loose body, resulting in painful mechanical symptoms such as catching and locking [12]. Treatment choice is based on skeletal maturity and lesion stability. The goals of the treatment are symptomatic relief and joint preservation. The terms “juvenile” and “adult” OCD are often used to distinguish patients with open and closed physes. However, these descriptions are cofounding, and patient designation in terms of physes status is more appropriate [13,14]. When a diagnosis is made in OCD patients with open physes (OCDO), healing can often be obtained via conservative therapy that mainly comprises the following: restricting sports activities, reduction in weight bearing on the affected side and using unloader braces [7]. Osteochondritis dissecans with closed physes (OCDC) rarely heals without surgery and has a worse prognosis [15]. OCD presenting in adults is thought to be a lesion that begins during skeletal immaturity but presents later in life and is predominantly reported among young men [16]. Treatment decision-making in OCD diagnosed in adults is similar to that of OCDC in adolescents and is based on lesion size and fragment stability. While OCDO has a better overall prognosis, it can require surgical treatment in the case of an unstable lesion or failure of conservative treatment [6,15].

A wide range of surgical methods is available in a surgeon’s armada. These include antegrade drilling (AD), microfracture (MF), lesion refixation, an osteochondral autograft transfer system (OATS) and autologous chondrocyte implantation (ACI) [17]. AD is indicated for stable lesions in skeletally immature patients. Reduction and internal fixation using metal or bioabsorbable screws is the most common surgical treatment for unstable but salvageable OCD fragments in both OCDO and OCDC. In the case of fragments that are considered unsalvageable, which are irreducible or show advanced cartilage degeneration, further decision making is based on the size of the cartilage defect. OATS is performed in defects <2–3 cm^2^, and ACI is generally the modality of choice in defects >3 cm^2^ [18].

Reports on long-term follow-up and athletic level following the surgical treatment of osteochondritis dissecans are scarce [19]. The aim of the present study is to report long-term clinical outcomes and activity level in sports after surgical treatment for OCD of the knee regardless of the growth plate’s condition. The authors of the present study hypothesized that surgical treatment produces and sustains good knee function with a high level of physical activity even in high-intensity sports that strain the knee. Furthermore, we hypothesize that patients with atypical localization OCD and those with larger/deeper osteochondral defects have a worse outcome than patients with typical localization and smaller/superficial defects.

## 2. Materials and Methods

**Figure 1:**
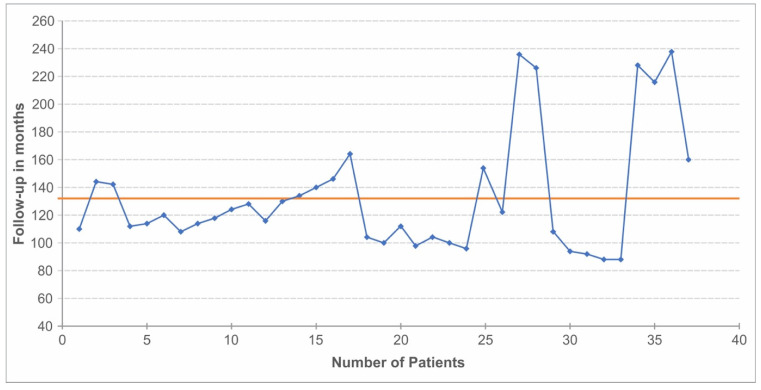
Patients’ follow-up duration. The time in months on the ordinate and the case number of the patients on the abscissa.

All patients aged 8 to 18 years who underwent surgery for knee OCD in Heidelberg University Hospital between 1993 and 2007 were included. Forty-five patients were in the initial cohort. Exclusion criteria included another surgery in the same knee for another reason or a knee injury/condition that would affect the outcome due to additional functional impairment of the joint. As a result, 2 of the 45 patients were excluded (tibial plateau fracture/patellar dysplasia). All patients were contacted via mail, email, or telephone. Six patients were lost to follow-up. A total of 37 patients made it to the final cohort: 30 male and 7 female patients. Twenty-one patients suffered from OCDO, and nine patients suffered from OCDC. In 7 patients, the physeal state at the time of surgery could not be determined due to a lack of preoperative imaging. The average patient age at the time of surgery was 14 (range 8–18) years. The BMI of all patients was recorded with a subgroup division of BMI < 25 kg/m^2^ and BMI ≥ 25 kg/m^2^. The average follow-up duration was 14 years (range 8–18) (Figure 1).

The study was conducted in accordance with the ethical standards outlined in the 1964 Declaration of Helsinki, which was revised in 2013. The study was approved by the local institutional review board (S-127) of Heidelberg University. All patients provided informed consent for participation in the study.

### 2.1. Defect Size, Depth, and Localization

Defect localization was divided into typical and atypical localizations to possibly examine a connection between lesion localization and outcome. The typical location of the defect corresponds to position 2B according to Cahill and Berg/Harding classifications [20,21]. Twenty-four of the 37 patients had defects in position 2B. In 13 patients, defect localization was considered atypical at any other site outside of 2B. These included 7 patients with OCD at the lateral femoral condyle and 6 patients with OCD at the patella. The ratio of typical to atypical localization was 1.8:1.

The size of the defect on the articular surface had been determined preoperatively using magnetic resonance imaging (MRI) and/or intraoperative findings. The patient cohort was subdivided into two groups: one with a defect size of <4 cm^2^ (*n* = 21) and the other with a defect size of ≥4 cm^2^ (*n* = 9). In 7 patients, no data about the defect size were available. Regarding the defect depth, patients were also divided into two groups based on the mean value (0.8 cm^2^, SD ± 0.35 cm^2^): the first group with a defect depth of <0.8 cm^2^ (*n* = 15) and the second group with a defect depth of ≥0.8 cm^2^ (*n* = 14). In 8 patients, no data regarding the defect depth were available.

### 2.2. Surgical Intervention and Revisions

The patients were primarily treated using different methods: 11 patients were treated with AD, 10 were treated with fragment refixation, 7 were treated with an MF, 5 were treated with ACI, and 1 was treated with OATS. Three patients received undetermined interventions. The choice of treatment was decided intraoperatively depending on the grade and size of the defect and if the lesion was salvageable or not. After an average duration of 40 months, 8 patients received secondary surgery due to insufficient cartilage regeneration of the defect and/or persistence of significant symptoms. In 2 cases, ACI was carried out after an initial AD and MF. Two cases of refixation were secondarily treated with MF. In 1 case, refixation was carried out following an OATS procedure. One case was revised using refixation following an initial ACI. In another 2 cases of ACI, a revision was carried out, involving cartilage resurfacing merely due to hypertrophy.

### 2.3. Subjective Knee Scores and Sports Questionnaires

The IKDC and Lysholm questionnaires were used to assess knee function. Pain intensity was assessed on a 0–10 point scale. Regarding physical activity, all patients received a previously unvalidated sports and medical history questionnaire [22,23]. A newly designed sports questionnaire was used to better compare previous data with newly collected results. The current sporting activity was evaluated and divided into knee-friendly sports and knee-straining sports, where repetitive peak loads or an increased load on the knee joint is expected [24,25]. An ordinal scale was used for activity and motivation. The duration and intensity (hours/week) of sports as well as membership in a sports club were also noted. 

### 2.4. Statistical Analysis and Comparison with Midterm Data

The same cohort was previously examined for the mentioned parameters after a median follow-up of 40 months. The outcome was compared with the final follow-up results. SPSS was used for further statistical evaluation. Normality was assessed using the Shapiro–Wilk test. In the case of normal distributions, the *t*-test was used; otherwise, the Kruskal–Wallis test or Mann–Whitney U test were used. Dichotomous classifications were calculated using the chi-square test. A *p*-value of <0.05 was considered statistically significant.

## 3. Results

### 3.1. Subjective Scores of Knee Function

**Figure 2:**
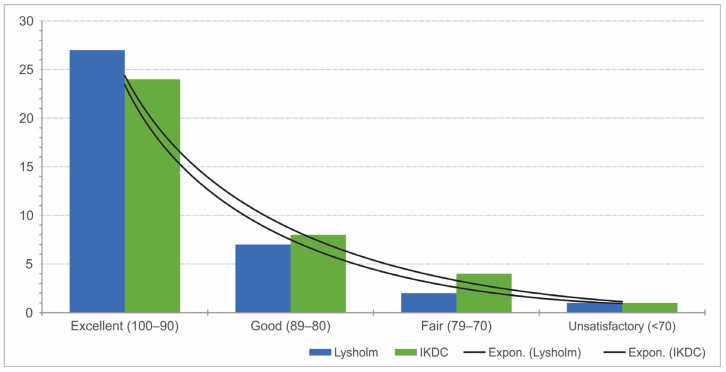
Display of the number of assessments according to the results in the bar chart.

With respect to the IKDC score, the mean value was 91.3 points (range: 64–100; SD ± 8.9). The mean Lysholm score was 91.7 points (range 64–100, SD ± 9). Overall, the long-term result of knee function was excellent on average. Twenty-four patients and 27 patients showed excellent results with in the IKDC and Lysholm scores, respectively. Only one patient showed a poor result on follow-up (Figure 2). 

**Figure 3:**
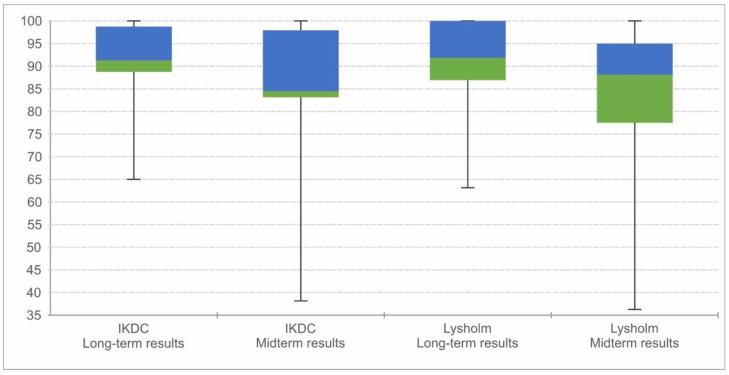
Display of the midterm and long-term overall results in a boxplot.

Only 1 patient still complained of constant pain while 11 patients reported pain in the knee after moderate to heavy loads. The rest of the patients (*n* = 25) were completely pain-free. The mean IKDC score at midterm follow-up was 84.4 (SD ± 134), and the Lysholm score was 87.7 (SD ± 14.2). Comparing the two outcomes, the mean scores increased during the long term with significant differences (IKDC *p*-value: 0.028/Lysholm *p*-value: 0.01) (Figure 3).

### 3.2. Results for OCDO and CODC

Patients who suffered from OCDO scored a mean IKDC value of 93 points and scored 94 points with respect to the Lysholm score. Patients who suffered from ACOD had a mean of 86 points with respect to the IKDC score (*p* = 0.068) and 85.5 points with respect to the Lysholm score (*p* = 0.034), thus showing a significant difference with respect to the Lysholm score in favor of OCDO.

### 3.3. Results of Defect Size, Depth, and Localization

The mean defect depth was 0.79 cm^2^. The patients were divided into two groups with a defect depth of <0.8 cm^2^ and ≥0.8 cm^2^. The IKDC score of the group with a defect depth of <0.8 cm^2^ was 94.5 points ± 5.8 and, with respect to the Lysholm score, it was 95.5 points ± 5.3 (Table 1). With a defect depth of ≥0.8 cm^2^, the mean IKDC was 87 ± 11.2 points, and the mean Lysholm was 87.2 ± 11.8. There was a significant increase (*p* = 0.023) in the IKDC of 7.5 points in patients with a defect depth of <0.8 cm^2^ compared to ≥0.8 cm^2^, and with respect to the Lysholm score (*p* = 0.015), it was 8.3 points. The results with typical localization had a mean value of 92.6 ± 9.8 points for IKDC and 93.3 ± 9.57 for the Lysholm score. In cases with atypical localization, the mean value for the IKDC was 89 points ± 6.2 (*p* = 0.283) and 89.4 ± 7.5 points (*p* = 0.251) for the Lysholm score. All results represent excellent or good subjective knee function with no significant differences in patients with typical and atypical defect localization (Table 1).

### 3.4. Results According to BMI

When evaluating the long-term function according to BMI, the results were not statistically significant (*p* > 0.05). Patients (*n* = 13) with a BMI of ≥25 kg/m^2^ achieved 90.6 ± 8.8 points with respect to the IKDC and 92.6 ± 7 points with respect to the Lysholm score, and patients (*n* = 24) with a BMI of <25 kg/m^2^ exhibited values of 91.60 ± 9 for IKDC and 91.25 ± 10 points with respect to the Lysholm score.

### 3.5. Sports

**Figure 4:**
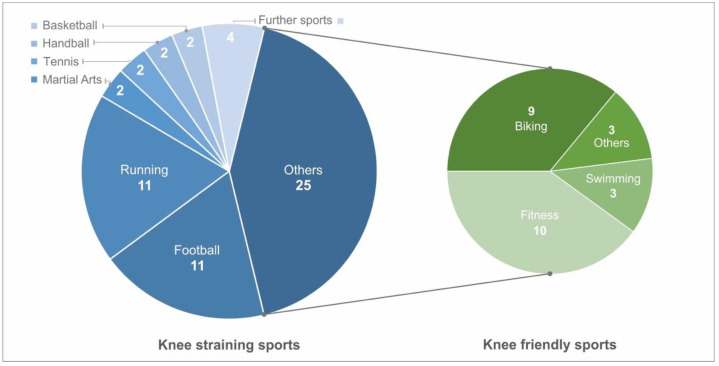
Pie chart representation of the sports practiced, divided into knee-straining and knee-friendly sports.

All except one patient reported playing sports occasionally or regularly. A total of 60 sports were observed on follow-up. These were divided into knee-friendly and knee-straining sports according to the load on the knee joint. In this study, 58% of the sports mentioned were classified as knee-straining. A total of 26 (72%) patients reported playing knee-straining sports, whereas 10 patients (28%) participated in knee-friendly sports (Figure 4). Out of the 60 sports activities reported as regularly carried out, 32 instances were carried out for less than 3 h per week. Twenty-three sports instances were carried out between 3 and 6 h per week. Only two patients reported performing a sport between 7 and 10 h per week, and two others reported spending more than 10 h a week on a sport.

**Figure 5:**
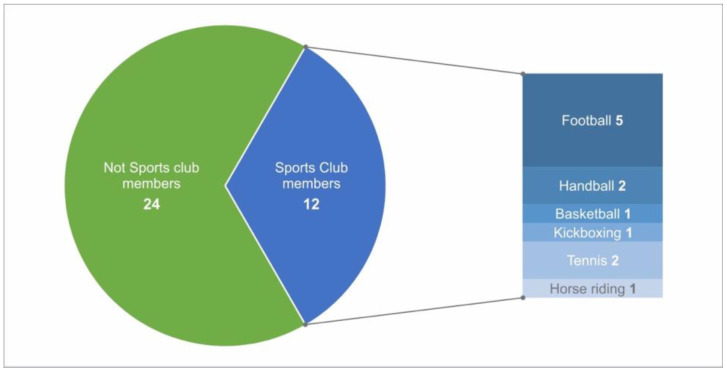
Pie chart representing the number of patients who are members of sport clubs.

**Figure 6:**
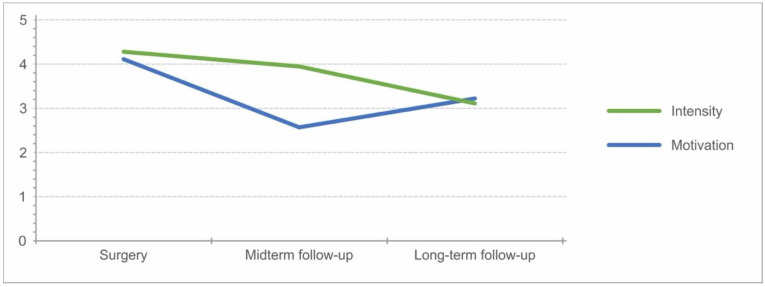
Development of intensity and motivation of patients starting from surgery until final follow-up.

The analysis of IKDC for patients participating in knee-straining sports resulted in significantly better results, with a mean value of 94 ± 6 (*p* = 0.04) and a Lysholm score of 95 ± 5.9 (*p* = 0.05), than those who performed knee-friendly sports or no sports at all. Twenty-four out of 36 patients being members of sport clubs (Figure 5). The development of intensity decreased throughout the follow-up, while motivation increased in the long-term after an initial decrease at midterm follow-up (Figure 6).

### 3.6. Results According to Surgical Intervention and after Revisions

**Figure 7:**
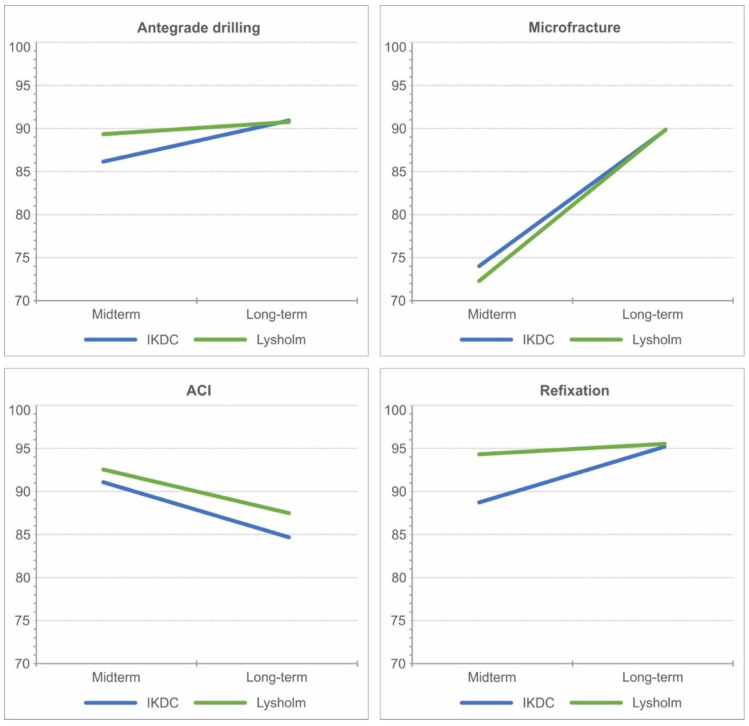
Display of the development of subjective knee scores after various interventions.

**Figure 8:**
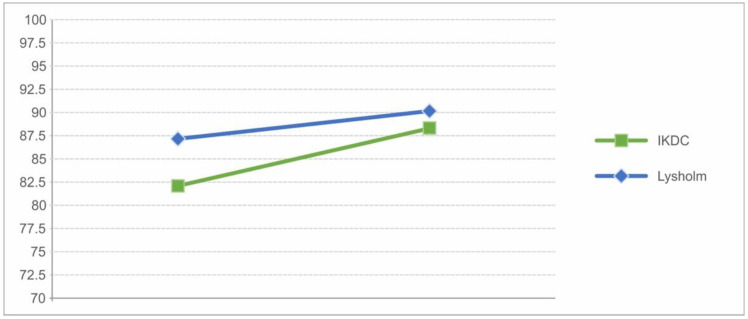
Display of the development of subjective knee scores following revisions.

The results of the last follow-up are displayed in Table 2. Figure 7 shows the improvement in subjective scores over time. Except for the ACI, all scores of surgical interventions improved in the long-term course, with MF showing the greatest improvement at 21 points in IKDC and 26.7 points in the Lysholm score. The other methods showed no significant differences and sustained good to excellent outcomes overall. The eight patients who received a revision surgery also showed a sustained improvement after their revision (Figure 8).

## 4. Discussion

The present cohort study reports a good long-term outcome with high athletic level up to 18 years following surgical treatment for OCD of the knee. The functional outcome was determined using subjective questionnaires with respect to IKDC and Lysholm scores as well as specially developed and not yet validated sports questionnaires to give a specific overview regarding the athletic strain on the knee joint. 

The physeal condition remains an important factor in the long-term outcome of surgically treated OCD and the treatment of OCD in general [26]. The present study also shows better long-term knee function in OCDO compared OCDC. Bruns et al. reported a similar difference in the Lysholm score of OCDO compared to OCDC with a follow-up of 10 and 20 years [27]. Goebel et al. reported a better radiological outcome in postoperative MRI for OCDO with a mean follow-up of 38 months [28]. Louisia et al. found significantly better clinical results in OCDO compared to the adult form, with an average follow-up of more than 11 years and with a good outcome in 50% compared to 70% of patients, respectively [29].

The present study shows no worsening of the outcome in the long-term compared to existing midterm data even after an almost tripled follow-up duration. On the contrary, an improvement in the outcome was observed. Husen et al. reported a similar sustained outcome following a fixation on long-term follow-up, suggesting that failure could occur relatively early after initial therapy, but results tend to remain stable after 5 years [30,31].

It has been reported that patients with OCD undergo TKA at a younger age than patients with primary osteoarthritis [32,33]. Regarding conversion surgery to total knee arthroplasty (TKA) in our cohort, none were reported during the long-term follow-up. We cannot, however, report on the progression of osteoarthritis since no radiological diagnostics were carried out on follow-up. High-level evidence exists and supports the early progression of osteoarthritis and conversion to TKA in knee OCD primarily in patients who received lesion excision and no further restorative interventions [19,34,35]. In cohorts with advanced OCD treated with refixation or in the case of unsalvageable lesions, the conversion rates to TKA remained very low with respect to ACI [31]. Hevesi et al. reported a TKA conversion rate of 3% in 95 patients with OCDO, more than half of which were treated operatively [36].

The return to sports following OCD is more crucial in the young population since athletes regularly performing sports that result in microtrauma to the knee are at increased risk for the development of OCD [1,37]. All but one patient in our cohort remained active in the long-term, which is reassuring. In the medical literature, there is an unfortunate lack of studies on the return-to-sport rate and intensity of physical activity following operative treatment for knee OCD. Husen et al. reported a good mean KOOS (sports and recreation) score of 80 points in his cohort, in which a total of 81 patients were treated via fragment refixation for OCD with an average follow-up of 11.3 years [30]. In a retrospective study carried out by Krych et al., patients treated with MF or OATs for symptomatic articular cartilage defects of the femoral condyles or trochlea had similar clinical outcomes after 5 years, but patients treated with OATS showed a superior level of athletic activity compared with those treated with MF [38]. However, most of these patients did not suffer from primary OCD, and the average age at the time of surgery was 30 years. We did not see a relevant difference between MF and other methods in our cohort. Regenerative procedures such as MF are more efficient in younger patients especially those with open physes. Cole et al. reported similar results and always used ACI as a revision procedure [39]. The ACI group in our cohort did not show a rapid improvement compared to other methods. However, it is important to consider that the maturation of the ACI method throughout the past years showed improving results, and third-generation ACI is significantly superior to previous generations [40]. The patients for whom revision surgery was indicated during the course also reported a significant improvement in knee function and knee scores.

Looking at further risk factors associated with a worse prognosis, no statistically significant differences in the subjective assessment of the knee joint were observed in relation to typical and atypical defect localizations, the size of the defect, or BMI at the time of surgery. However, we identified the increased depth to be a risk factor. So far, there has been no indication in available medical literature that defect size affects the results of surgically treated OCD. Husen et al. found that atypical localization (lateral femoral condyle) is associated with an increased rate of revisions in his cohort, but the defect depth was not evaluated [20]. Regarding defect size, the operative technique is tailored accordingly. Due to the variety and associated stage-dependent therapy options, making a definitive statement about the prognosis depending on the defect area in our cohort remains difficult.

## 5. Limitations

The weaknesses in the present study include a lack of imaging studies that assess chondral and osseous conditions on long-term follow-up. Some preoperative imaging was also missing. As a result, we could not define the defect characteristics and physeal state in all patients. Furthermore, the intraoperative characteristics of stable/unstable and salvageable/unsalvageable osteochondral lesions are lacking due to data unavailability. This could have been an interesting addition. Regarding our cohort, six patients were lost to follow-up since they did not respond. It is unclear how these patients are doing or if they even received revision surgery in another institute. Our patients were treated using various interventions. In a few cases, we could not pinpoint the exact surgery that was carried out. A statement regarding the superiority of one surgical intervention over the other is thus weakened.

## 6. Conclusions

All therapy methods examined, whether revascularizing or reconstructive, achieved good to very good results with a high athletic level on the long-term, even when performing knee-straining sports. Individual treatment options should be stage-dependent. Nevertheless, refixation of osteochondral fragments should be preferred, if possible. A gradual improvement is likely and can also be expected from mid to long-term periods following surgery. The depth of the defect and the condition of the physes are relevant prognostic factors.

## Figures and Tables

**Table 1:** Summary of the results of the scores according to defect localization, defect size, and defect depth. * refers to significant *p*-values.

		N	Mean	SD	Minimum	Maximum	*p*-Value
Localisation	Typical	24	92.6	9.8	64.7	100	0.283
IKDC	Atypical	13	89.1	6.2	78.2	100	
Localisation	Typical	24	93.3	9.6	63.0	100	0.251
Lysholm	Atypical	13	89.4	7.5	75.0	100	
Defect size	<4 cm^2^	21	90.6	9.7	64.7	100	0.92
IKDC	≥4 cm^2^	9	92.4	8.9	73.6	100	
Defect size	<4 cm^2^	21	91.5	10.5	63.0	100	0.71
Lysholm	≥4 cm^2^	9	92.6	8.3	80.0	100	
Defect depth	<0.8 cm^2^	15	94.5	5.8	79.3	100	0.023 *
IKDC	≥0.8 cm^2^	14	87.1	11.1	64.7	100	
Defect depth	<0.8 cm^2^	15	95.5	5.3	83.0	100	0.015 *
Lysholm	≥0.8 cm^2^	14	87.2	11.8	63.0	100	

**Table 2:** Subjective knee results of various interventions.

Surgical Method	N	Mean	SD	Minimum	Maximum
Antegrade drilling	10				
IKDC		91.3	10.3	65	100
Lysholm		91	10.8	63	100
Microfracture	8				
IKDC		89.4	11.5	73	100
Lysholm		89.4	12.4	70	100
ACI	6				
IKDC		85.2	8.3	74	97
Lysholm		87.3	8.2	81	100
Refixation	9				
IKDC		94.8	4.1	87	100
Lysholm		95.7	3.2	93	100

## Data Availability

The data presented in this study can be made available upon reasonable request from the corresponding author.

## Abbreviations

OCD	Osteochondrosis dissecans
OCDO	Osteochondritis dissecans with open physes
OCDC	Osteochondritis dissecans with closed physes
MFC	Medial femoral condyle
OATS	Osteochondral autograft transfer system
AD	Antegrade drilling
MF	Microfracture
MRI	Magnetic resonance imaging
ACI	Autologous chondrocyte implantation
TKA	Total knee arthroplasty
